# Identification of mutant gene for Black crystal coat and non-allelic gene interactions in *Neogale vison*

**DOI:** 10.1038/s41598-022-14079-z

**Published:** 2022-06-21

**Authors:** Andrey D. Manakhov, Maria Yu. Mintseva, Lev I. Uralsky, Tatiana V. Andreeva, Oleg V. Trapezov, Evgeny I. Rogaev

**Affiliations:** 1 Department of Genetics, Centre for Genetics and Life Science, Sirius University of Science and Technology, 354340 Sochi, Russia; 2 Laboratory of Evolutionary Genomics, Department of Genomics and Human Genetics, Vavilov Institute of General Genetics, Russian Academy of Sciences, 119333 Moscow, Russia; 3 Centre for Genetics and Genetic Technologies, Faculty of Biology, Lomonosov Moscow State University, 119192 Moscow, Russia; 4 Department of Animals and Human Genetics, Institute of Cytology and Genetics, Siberian Branch of the Russian Academy of Sciences, 630090 Novosibirsk, Russia; 5 Novosibirsk State University, 630090 Novosibirsk, Russia; 6 Department of Psychiatry, UMass Chan Medical School, Worcester, MA 01604 USA

**Keywords:** Animal breeding, Genetics, Comparative genomics

## Abstract

Sable (*Martes zibellina*) and American mink (*Neogale vison*) are valuable species characterized by a variety of coat colour produced on fur farms. Black crystal fur phenotype is Mendelian codominant trait: heterozygous animals (*C*^*r*^*/* +) have white guard hairs scattered predominantly on the spine and the head, while homozygous (*C*^*r*^*/C*^*r*^) minks have coats resembling the Himalayan (*c*^*h*^*/c*^*h*^) or white Hedlund (*h/h*) types. It is one of the most recent of more than 35 currently known phenotypic traits of fur colour in American mink. Black crystal fur phenotype was first described in 1984 in the Russian population of mink, which had undergone selection for domestic defensive response to humans. Here, we performed whole-genome sequencing of American mink with *C*^*r*^*/C*^*r*^ phenotype. We identified a missense mutation in the gene encoding the α-COP subunit of the COPI complex (COPA). The COPI complex mediates retrograde trafficking from the Golgi system to the endoplasmic reticulum and sorting of transmembrane proteins. We observed an interaction between a newly identified mutation in the *COPA* gene and a mutation in the microphthalmia-associated transcription factor (*MITF*), the latter mutation led to the formation of the white Hedlund (*h/h*) phenotype. Double heterozygotes for these mutations have an entirely white coat and a black-eyed phenotype similar to the phenotype of *C*^*r*^*/C*^*r*^ or *h/h* minks. Our data could be useful for tracking economically valuable fur traits in mink breeding programs to contribute to global fur production.

## Introduction

More than a century of artificial selection for American mink (*Neogale vison*, previously known as *Neovison vison*^1^) has resulted in the emergence of a wide spectrum of coat colours. By the classical genetic analysis presents of 35 mutations affecting fur colour were predicted^2,3^. However, to date, only 8 of them have been linked with specific genes and DNA mutations^4–9^.

**Figure 1:**
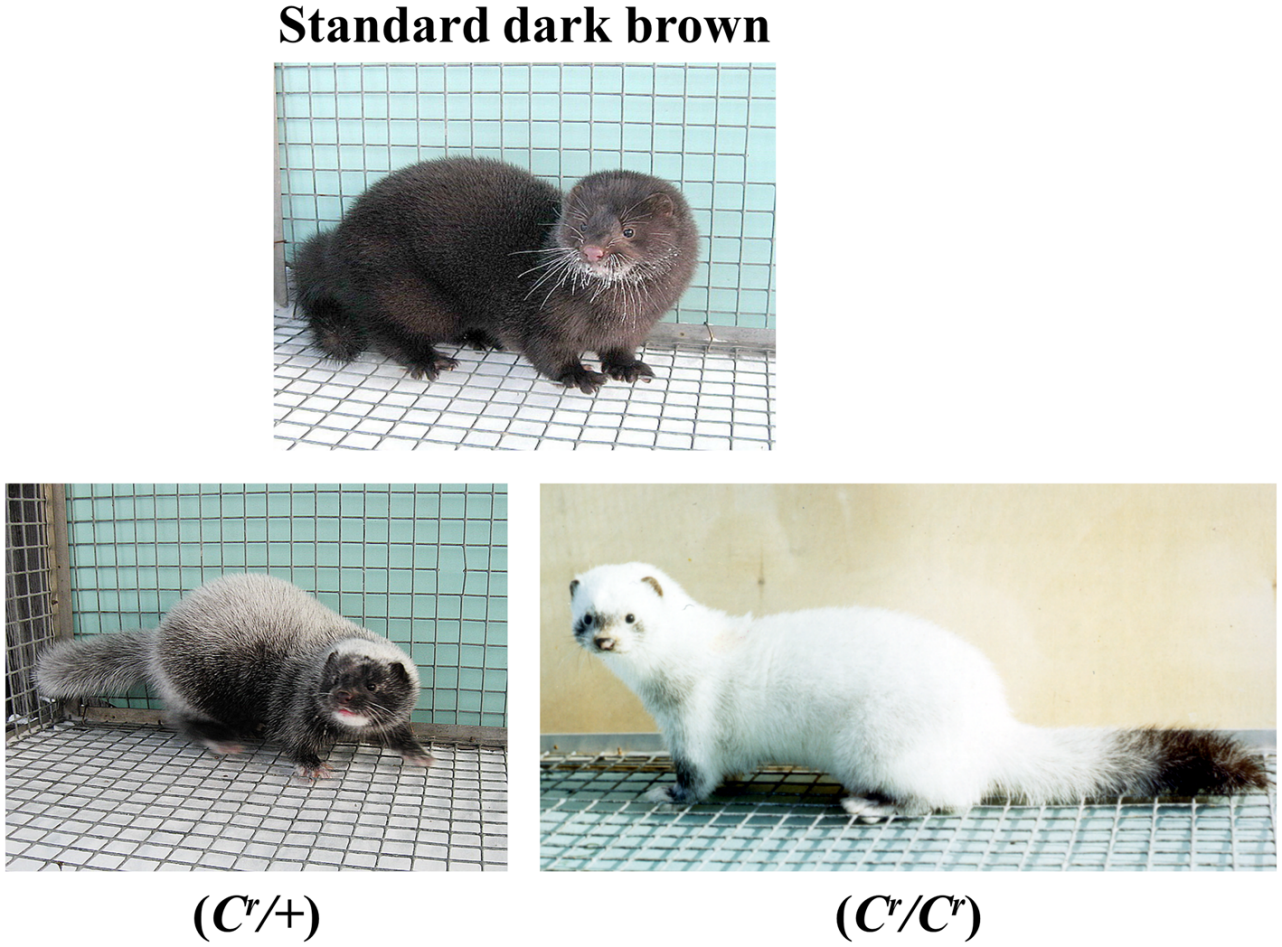
American minks with the standard dark brown, Black crystal heterozygous (*C*^*r*^*/* +) and Black crystal homozygous (*C*^*r*^*/C*^*r*^) phenotypes.

Black crystal is one of the most recent mink colour mutations and was first described in 1984 in a mink population of standard dark brown animals (Fig. 1) undergoing selection for domestic defensive reaction towards human in the Experimental Fur Farm of the Institute of Cytology and Genetics (Novosibirsk, Russia). The Black crystal phenotype was found to be inherited as Mendelian codominant trait: heterozygous animals (*C*^*r*^*/* +) have completely white guard hairs scattered predominantly on the spine and the head, and “white hat” is the marker trait of this phenotype (Fig. 1). Homozygous (*C*^*r*^*/C*^*r*^) minks have coats resembling the Himalayan type, with pigmented tips of the face, tail, and legs (Fig. 1)^10^. However, the number of pigmented guard hairs in *C*^*r*^*/C*^*r*^ animals is low, and during the maturation process, animals may produce a completely white phenotype similar to Hedlund white (*h/h*) but with no hearing defects. The eyes of both *C*^*r*^*/* + and *C*^*r*^*/C*^*r*^ minks are dark brown, similar to those of standard minks.

The goal of this study was investigation of the genetic mechanism determining the Black crystal phenotype. Here we performed the first whole-genome sequencing of American mink with a completely white (expected to be homozygous for the Black crystal mutation (*C*^*r*^*/C*^*r*^)) fur colour and whole-genome analysis in combination with 8 genomes of minks with other coats colour (Table 1).

**Table 1:** Results of sequencing of American mink genomes. Statistics were calculated using samtools^11^ and Picard software. The American mink genome (NNQGG.v01) was used as a reference.

Sample	Colour name	Colour symbol	Mapped %	Duplicates %	Coverage
mink_4-131	Black crystal*	*C*^*r*^*/C*^*r*^	98.06	0.86	9.37
mink_3-247^8^	Standard dark brown	+ */* +	98.70	2.72	7.07
mink_3-261^8^	Standard dark brown	+ */* +	97.89	4.23	9.08
mink_3-265^8^	Standard dark brown	+ */* +	98.07	2.30	8.53
mink_7-331^9^	Moyle	*m/m*	98.15	0.70	8.11
mink_7-317^9^	Violet	*a/a m/m p/p*	98.46	8.53	40.32
mink_0-329^8^	Silverblue	*p/p*	98.30	3.13	5.43
mink_1-663^8^	Silverblue	*p/p*	97.14	11.82	5.73
mink_9-431^8^	Silverblue	*p/p*	97.82	4.28	5.17

*Completely white animal, with no hearing defects.

## Results

**Figure 2:**
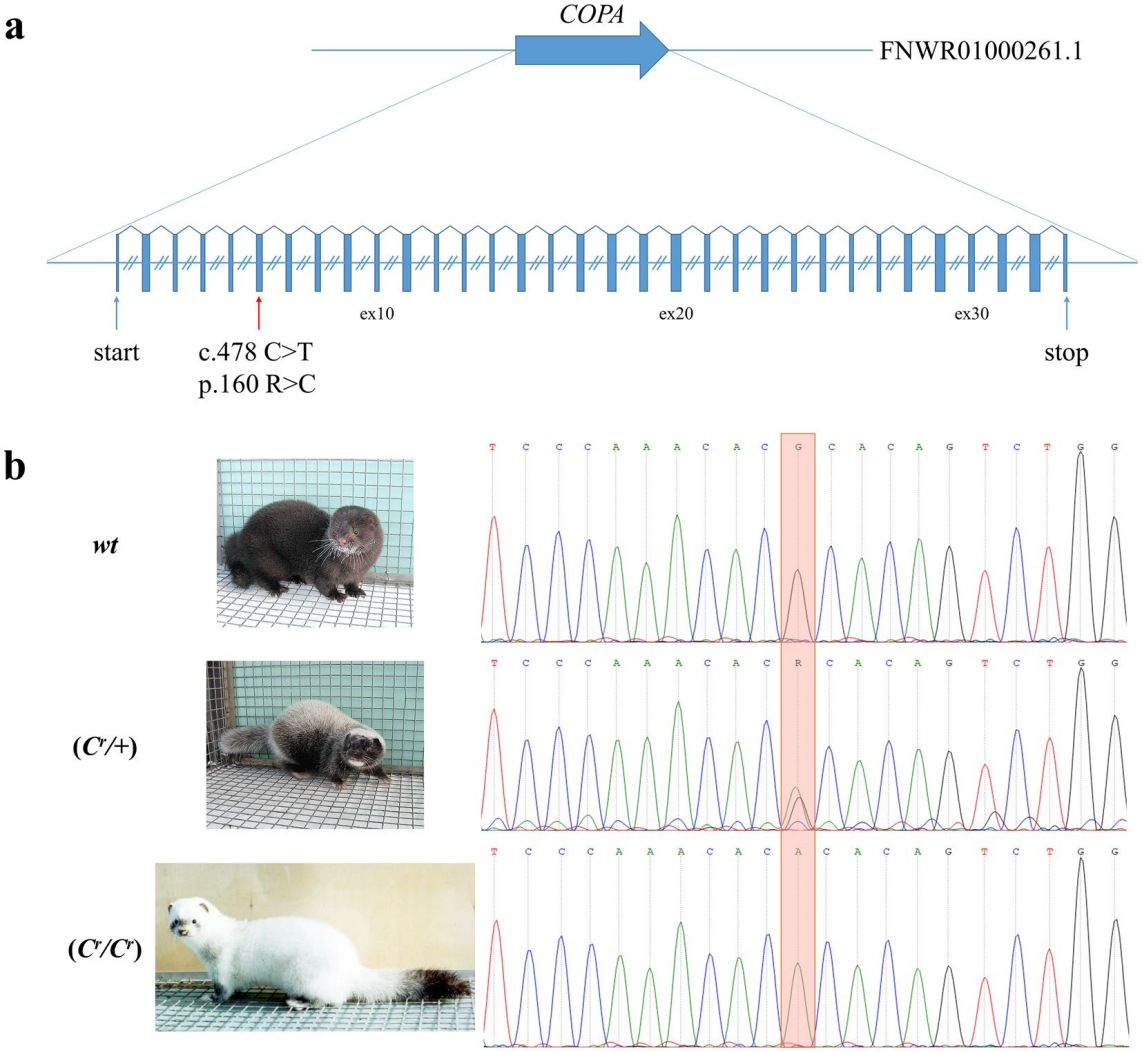
*COPA*^*Cr*^ mutation. (**a**) Structure of the mink *COPA* gene. The red arrow indicates the *COPA*^*Cr*^ mutation. Dotted boxes indicate 5’- and 3’-UTRs. Equal introns sizes are shown for simplification. (**b**) An electrophoregram of Sanger sequencing for *COPA* gDNA exon 6. The orange frame is the *COPA* c.478 C>T mutation in Black crystal animals.

Based on genomic data (Table 1) we identified 90 450 homozygous genetic variations in mink_4-131 (*C*^*r*^*/C*^*r*^) that were not homozygous or heterozygous in any standard dark brown, silverblue (*p/p*), moyle (*m/m*) and violet (*a/a m/m p/p*) animals (Supplementary Data 1). Then we prioritized for further analysis 176 variations that were observed in protein encoding regions (gene exons) and splicing sites (Supplementary Data 3). The genes bearing such variations were analyzed further for their direct or indirect potential involvement in the regulation of the pigmentation (Supplementary Data 3). Ultimately, we identified a single-nucleotide variation (FNWR01000261.1:4876673 G/A (*COPA* c.478 C>T), hereinafter referred to as *COPA*^*Cr*^) at the sixth exon of the coatomer protein complex subunit alpha gene (*COPA*) (Fig. 2).

The *COPA*^*Cr*^ mutation leads to amino acid substitution COPA p.Arg160Cys is a highly conserved among mammalian WD40 repeat motif of the COPA protein (Supplementary Fig. 1), and this substitution is predicted to be “probably damaging” by PolyPhen^12^. The mutation was heterozygous in all tested minks with a Black crystal phenotype (*C*^*r*^*/* +). However, not all white animals with black eyes, which were expected to be homozygous for the Black crystal mutation (*C*^*r*^*/C*^*r*^), have the *COPA*^*Cr*^ mutation in the homozygous state. No homozygous or heterozygous *COPA*^*Cr*^ mutation was observed in minks with other coat colour phenotypes (Table 2).

**Table 2:** Results of *COPA*^*Cr*^ and *MITF*^*h*^ genotyping in American mink. The *p*-value is 0.00023 for association of the T allele of *COPA*^*Cr*^ with Black crystal fur colour (OR = 218; 95% CI 12.45–3825.13).

Coat colour name	Coat colour symbol	Genotype *COPA*^*Cr*^	Genotype *MITF*^*h*^	Number of animals
Black crystal*	*C*^*r*^*/* +	T/C	G/G	10
Black crystal**	*C*^*r*^*/C*^*r*^	T/T	G/G	3
		T/T	G/A	1
		T/C	G/A	3
Standard dark brown	+ */* +	C/C	G/G	25
Violet	*a/a m/m p/p*	C/C	G/G	1
Royle pastel	*b/b*	C/C	G/G	1
Hedlund white	*h/h*	C/C	A/A	1
Moyle	*m/m*	C/C	G/G	1
Silverblue	*p/p*	C/C	G/G	3
Shadow Silverblue	*S*^*h*^*/* + *p/p*	C/C	G/G	1
Black cross	*S/* +	C/C	G/G	1

*Animals with white guard hairs on the spine and head.

**Completely white animals, with no hearing defects.

Previously, we identified that the Hedlund white phenotype in minks with black eyes is a result of a homozygous MITF-M c.33 + 1 G>A mutation (hereinafter referred to as *MITF*^*h*^)^8^. We hypothesized that white minks with black eyes possessing only one Black crystal allele *C*^*r*^ may have a Hedlund mutation, which leads to a similar white coat and black-eyed phenotype. We found that all white coat and black-eyed minks with the heterozygous *COPA*^*Cr*^ mutation additionally had the *MITF*^*h*^ mutation in the heterozygous state. Moreover, all tested standard dark brown minks, as well as minks with other colour coats, were not double or single heterozygotes for these mutations (Table 2).

Considering all the data, we propose that the cumulative effect of the *COPA*^*Cr*^ mutation underlying the Black crystal phenotype (*C*^*r*^*/* +) and with the *MITF*^*h*^ heterozygous mutation resulted in the white coat and black-eyed mink phenotype.

## Discussion

In the present study, we described a mutation in the *COPA* gene, which produces a Black crystal coat phenotype in American mink.

The *COPA* gene encodes the α-COP subunit of the heptameric COPI complex (α/β/β’/γ/δ/ε/ζ). COPI mediates retrograde trafficking from the Golgi to the endoplasmic reticulum and sorting of transmembrane proteins^13^. Previously, several mutations in genes encoding different COPI subunits have been reported to cause pigmentation aberrations in zebrafish (*copa*, *copb1*, *copb2*)^14^, mouse (*Copd*)^15^, and cattle (*COPA*)^16,17^. However, the exact role of the COPI complex in pigmentation processes remains obscure.

The *COPA*^*Cr*^ mutation results in amino acid substitution p.Arg160Cys at the end of the fourth highly conserved WD40 repeat motif of the COPA protein. WD40 repeats are involved in the binding of the COPI complex with dilysine motifs (KKxx, KxKxx) of cargo proteins^13,18^. Interestingly, the same amino acid substitution in the COPA protein was previously shown to transform the dominant black phenotype (caused by an *MC1R*^*D*^ mutation resulting in a constitutively active MC1R receptor) to the dominant red coat colour in Holstein cattle^16,17^. Hair pigment analysis and expression studies have shown downregulation of melanogenic genes and a switch in pigment production towards pheomelanin in cattle with the dominant red phenotype (*MC1R*^*D*^*/MC1R*^*D*^, *COPA*^*Cr*^*/COPA*^+^) compared to dominant black cattle (*MC1R*^*D*^*/* + , *COPA*^+^*/COPA*^+^)^16,17^. To the best of our knowledge, our study is the first to report a *COPA* gene mutation in animals with no mutations in the *MC1R* gene. Based on previous studies^16,17^, we hypothesized that the *COPA*^*Cr*^ mutation may potentially lead to defects in the trafficking of proteins required for melanogenesis or its regulation.

Interestingly, we observed that double heterozygotes (*COPA*^*Cr*^*/* + , *MITF*^*h*^*/* +) have a completely white coat and black-eyed phenotype similar to the phenotype of *C*^*r*^*/C*^*r*^ or *h/h* minks but with normal hearing, unlike the latter. *COPA*^*Cr*^*/* + animals have completely white guard hairs scattered predominantly on the dorsal side^10^, while *MITF*^*h*^*/* + minks have white spots located predominantly on the ventral side of the body^19^. Presumably, fusion of depigmentation zones in double heterozygotes (*COPA*^*Cr*^*/* + , *MITF*^*h*^*/* +) results in the formation of the completely white coat; however, no other interactions between *COPA* and *MITF* gene mutant products can be excluded.

Initially, the Black crystal mutation originated in a mink population undergoing long-term selection for domestic defensive reaction towards man and was suggested to be involved in animal behaviour phenotypes^10^. Previously, a mutation in the mouse *Copd* gene, which encodes another subunit of the COPI complex, was demonstrated to lead to Purkinje cell degeneration and ataxia^15^. Moreover, COPI vesicles were reported to potentially act as authentic transport vehicles within axons and dendrites^20^. Thus, Black crystal minks may be a new model to understand the roles of COPA and COPI in the nervous system.

Taken together, our present study adds *COPA* gene to the list of mapped mink colour genes^4–9^ and provides valuable data that can contribute to improving global mink fur production through selective breeding programmes. Furthermore, considering the potential role of *COPA* gene mutation in nervous system functions, Black crystal minks may serve as a new unique model for studies of animal behaviour mechanisms.

## Methods

All methods were carried out in accordance with relevant guidelines and regulations for laboratory work as well as ARRIVE guidelines. The local Ethics Committee of the Institute of Cytology and Genetics approved the study protocols.

Black crystal (*C*^*r*^*/* + 10 individuals and *C*^*r*^*/C*^*r*^ 7 individuals), shadow silverblue (*S*^*h*^*/* + *p/p* 1 individuals), black cross (*S/* + 1 individual), violet (*a/a m/m p/p* 1 individual), Royle pastel (*b/b* 1 individual), Hedlund white (*h/h* 1 individual), moyle (*m/m* 1 individual), silverblue (*p/p* 3 individuals) and standard dark brown (25 individuals) farm-bred American minks were maintained in the Experimental Fur Farm of the Institute of Cytology and Genetics (Novosibirsk, Russia). Collected tissues were rapidly dissected and frozen in liquid nitrogen and then stored at − 70 °C until DNA extraction.

Genomic DNA from mink tissues was extracted using QIAGEN Mini Spin Columns following the manufacturer’s protocol (QIAGEN, Germany). Library preparation from the DNA of completely white animal (mink_4-131), which were expected to be homozygous for the Black crystal mutation (*C*^*r*^*/C*^*r*^), was performed with the TruSeq PCR Free Kit (Illumina, USA) following the manufacturer’s protocol. Library validation was performed with an Agilent 2100 Bioanalyzer with a DNA High Sensitivity chip (Agilent, USA) and quantified with qPCR using a KAPA Library Quantification Illumina Kit protocol (KAPA Biosystems, USA). The paired-end library was sequenced in 2 × 76 and 2 × 101 cycles with the Illumina RapidRun SBS v2 kit (Illumina, USA), and in 2 × 101 cycles with the Illumina TruSeq SBS v3 kit (Illumina, USA) on a HiSeq 2000/2500 sequencer (Illumina, USA) at the Vavilov Institute of General Genetics RAS (Moscow, Russia).

Additionally, we used whole-genome sequencing data from 3 standard dark brown, 3 silverblue, 1 moyle and 1 violet mink from our previous studies (Table 1)^8,9^.

The resulting reads were mapped to the mink genome (NNQGG.v01) using a BWA-MEM algorithm (bwa v.0.7.13-r112)^21^. Duplicate reads were detected with the MarkDuplicates algorithm from picard-tools v.1.133 (http://broadinstitute.github.io/picard) and excluded from further analysis.

Genetic variants in sequenced mink genomes were predicted using the Genome Analysis Toolkit (GATK) HaplotypeCaller package version 4.0^22^.

To detect the genetic factor underlying the Black crystal phenotype, we selected homozygous variants with a depth of coverage greater than 2 in the mink_4-131 genome that were not homozygous or heterozygous in all other colour phenotypes (standard dark brown, silverblue, moyle and violet). Sample mink_4-131 has completely white fur and normal hearing and was expected to be homozygous for the Black crystal mutation (*C*^*r*^*/C*^*r*^).

Annotation and effect prediction of selected variants were performed in SnpEff^23^ using mink genome annotation.

We performed Sanger sequencing to validate the selected mutation. Primers for PCR amplification were designed in Primer3 software (Table 3), and PCR was performed with GenPack PCR Core (Isogen, Russia). Resultant amplicons were cleaned with a Cleanup Standard Kit (Evrogen, Russia) and processed with the BigDye Terminator v3.1 Cycle Sequencing Kit (Applied Biosystems, USA) following the manufacturer’s protocol. Probes were purified using a DyeEx 2.0 Spin Kit (QIAGEN, Germany) and sequenced in a 3730xl DNA Analyzer (Applied Biosystems, USA).

**Table 3:** Primer sequences used for cDNA and gDNA amplification.

Primer name	Primer sequence	Expected amplicon size (bp)	Annealing t (°C)
*gDNA COPA ex 6 F*	TTCCTCAACAATCCGCTAAC	506	58
*gDNA COPA ex 6 R*	TCAGAGGAAAGAAGGGGACT		
*gDNA MITF ex 1 M F*	CTTCTCTATGCCCGTCAGTC	368	58
*gDNA MITF ex 1 M R*	GAACAGGAGCTGATGGAGAG		

## Supplementary Information

Supplementary Information.

Supplementary Data.

Supplementary Figure 1.

## Data Availability

The datasets generated during the current study were deposited into the NCBI SRA database and can be accessed with the BioProject accession number PRJNA660737 (https://www.ncbi.nlm.nih.gov/sra/PRJNA660737).
