# Supplementary material for: Identification of mutant gene for Black crystal coat and non-allelic gene interactions in Neogale vison
Source: Sci Rep. 2022 Jun 21;12:10483. doi: 10.1038/s41598-022-14079-z (PMC9213499; doi:10.1038/s41598-022-14079-z)
Supplement: Supplementary file 1 — Supplementary Information. [file 41598_2022_14079_MOESM1_ESM.pdf]

Identification of mutant gene for Black crystal coat and non-allelic gene interactions in *Neogale vison*

Andrey D. Manakhov[1, 2, 3] (orcid: 0000-0002-5163-8747)

Maria Yu. Mintseva[2] (orcid: 0000-0003-4613-471X)

Lev I. Uralsky [1, 2] (orcid: 0000-0002-5565-7961)

Tatiana V. Andreeva[2, 3]

Oleg V. Trapezov[4,5]

Evgeny I. Rogaev[1, 2, 3, 6] *

1) Department of Genetics, Centre for Genetics and Life Science, Sirius University of Science and Technology, 354340 Sochi, Russia
2) Laboratory of Evolutionary Genomics, Department of Genomics and Human Genetics, Vavilov Institute of General Genetics, Russian Academy of Sciences, 119333 Moscow, Russia
3) Centre for Genetics and Genetic Technologies, Faculty of Biology, Lomonosov Moscow State University, 119192 Moscow, Russia
4) Department of Animals and Human Genetics, Institute of Cytology and Genetics, Siberian Branch of the Russian Academy of Sciences, 630090 Novosibirsk, Russia
5) Novosibirsk State University, 630090 Novosibirsk, Russia
6) Department of Psychiatry, UMass Chan Medical School, Worcester, MA 01604, USA

* Corresponding author (rogaev@vigg.ru)

Supplementary Figure 1. Alignment of the fourth highly conserved WD40 repeat motif of the COPA protein (aa 126-164 for *Neogale vison*) among 86 mammals. The orange arrow indicates p. Arg160Cys variation, identified in Black crystal (C_r) minks.

Supplementary Data 1. VCF with identified 90 450 homozygous genetic variations in mink_4-131 (C^r/C^r) that were not homozygous or heterozygous in any standard dark brown, silverblue (*p/p*), moyle (*m/m*) and violet (*a/a m/m p/p*) animals.

Supplementary Data 2. VCF with 176 variations that were observed in protein encoding regions (gene exons) and splicing sites selected from 90 450 homozygous genetic variations in mink_4-131 (C^r/C^r).

Supplementary Data 3. VCF with 7 homozygous genetic variations in mink_4-131 (C^r/C^r) that were observed in protein encoding regions of genes that involved in the regulation of pigmentation.
