# Supplementary figures and images for: Identification of mutant gene for Black crystal coat and non-allelic gene interactions in Neogale vison
Source: Sci Rep. 2022 Jun 21;12:10483. doi: 10.1038/s41598-022-14079-z (PMC9213499; doi:10.1038/s41598-022-14079-z)

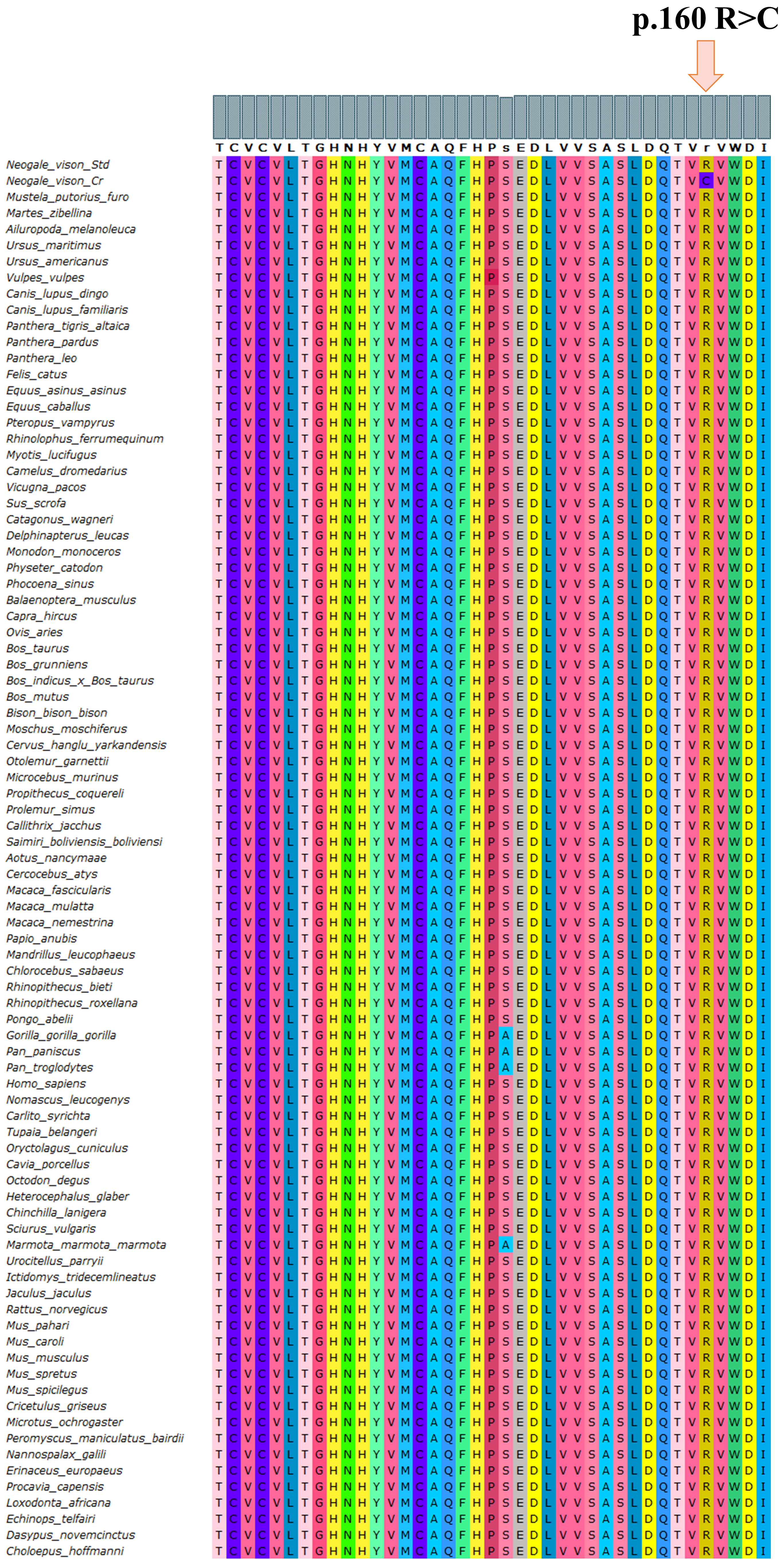

Supplement: Supplementary file 3 — Supplementary Figure 1. [file 41598_2022_14079_MOESM3_ESM.tif]
